# Sequential changes in the gene expression profile of murine retinal progenitor cells during the induction of differentiation

**Published:** 2009-10-20

**Authors:** Ping Gu, Jing Yang, Jinmei Wang, Michael J. Young, Henry Klassen

**Affiliations:** 1 Gavin Herbert Eye Institute, Department of Ophthalmology, School of Medicine, University of California, Irvine, Orange, CA; 2 Department of Ophthalmology, Shanghai Ninth People's Hospital, School of Medicine, Shanghai Jiaotong University, Shanghai, China; 3 Schepens Eye Research Institute, Department of Ophthalmology, Harvard Medical School, Boston, MA

## Abstract

**Purpose:**

Following transplantation, cultured retinal progenitor cells (RPCs) integrate into the diseased host retina and exhibit morphologies and markers indicative of local cellular phenotypes. In vitro analysis of cultured RPCs allows detailed examination of marker gene expression during the initial phase of differentiation and can provide insight into the variables influencing this process.

**Methods:**

Using cultured murine RPCs, this study compares the effects of fetal bovine serum (FBS) with those of ciliary neurotrophic factor (CNTF), individually or in combination with epidermal growth factor (EGF). Differentiation was assessed by way of the relative expression of 17 genes using quantitative PCR (qPCR) at five time points over a seven-day period.

**Results:**

Both CNTF and FBS rapidly altered the gene expression of RPCs, with very marked upregulation of glial fibrillary acidic protein (*GFAP*; FBS>CNTF) and marked down-regulation of the proliferation marker *K_i_-67*, consistent with the induction of differentiation. The evidence supports a preponderantly pro-glial influence for both the FBS and CNTF, however, neuronal markers were also upregulated to a lesser extent. Immunocytochemistry confirmed subpopulations labeling with neuronal markers, including rhodopsin. In the presence of sustained EGF stimulation, the differentiating influences of both FBS and CNTF remained perceptible as transient peaks of relative gene expression, but were markedly diminished overall.

**Conclusions:**

This study shows that it is possible to compare the relative efficacy of in vitro differentiation protocols using murine RPCs and qPCR. The differentiating influences of both serum and CNTF were confirmed, but shown to be powerfully moderated by EGF. This suggests that EGF withdrawal is the dominant feature of these differentiation protocols and that exposure to either serum or CNTF is insufficient to irreversibly commit a cultured RPC population to terminal differentiation unless accompanied by concomitant cessation of mitogenic stimulation.

## Introduction

The permanent loss of retinal neurons underlies several common blinding conditions, most of which are effectively untreatable. The idea of replacing lost neurons in the diseased retina has long been entertained as a speculative, yet ostensibly unrealistic, notion up until the advent of the current area of retinal transplantation studies. The demonstration that grafts of embryonic retinal tissue could survive and form functional connections [1,2] with the host visual system in mammals led to renewed investigations of the potential for orthotopic graft-to-host integration within the retina. With these studies, the limitations of immature retinal tissue grafts gradually became apparent [3]. In particular, it emerged that a method was needed for achieving the widespread integration of grafted cells. This result was first reported following the use of hippocampal progenitor cells as donor cells [4,5].

The work with brain-derived progenitor cells showed that these cells were capable of cytoarchitectural integration and morphological development consistent with retinal neurons, including photoreceptors [5], and survived without the need for exogenous immune suppression [6], however, the evidence for expression of photoreceptor markers was quite limited [7]. Together, these findings suggested that brain progenitors are developmentally restricted from fully differentiating into photoreceptors under the conditions tested. To explore whether more complete photoreceptor differentiation could be achieved using an alternate cell type, progenitor cells were subsequently cultured from the immature retina. These retinal progenitor cells (RPCs) not only integrated into the outer nuclear layer (ONL) of the retina but also expressed the photoreceptor markers, recoverin and rhodopsin, and were associated with partial preservation of light sensitivity in animals with photoreceptor degeneration [8]. Similar results have subsequently been obtained with uncultured precursor cells [9] and following differentiation of embryonic stem cells [10,11], lending additional support to the basic premise of retinal cell replacement through cell transplantation.

Viewed as a potential therapeutic strategy, the replacement of retinal cell types using stem, progenitor, or precursor cells presupposes that the grafted cells not only integrate but also differentiate completely and along the appropriate lineages. The availability of cultured RPCs now affords a greater opportunity for systematic investigation of the differentiation process in vitro. Differentiation conditions that are among the most widely applied to retinal explants and RPC cultures include the substitution of mitogenic growth factors with either fetal bovine serum (FBS) or ciliary neurotrophic factor (CNTF), the later representing an example of a defined, serum-free condition.

The differentiating influence of serum has long been appreciated and neural stem and progenitor cells were only successfully propagated in culture once a serum-free method had been established [12]. CNTF has not only been implicated in retinal development, but has also been shown to influence RPCs [13] and retinal explants [14], and has found an additional role in the neuroprotection of degenerating photoreceptors [15-17]. FBS and CNTF have both been reported to result in glial specification and induction of glial fibrillary acid protein (*GFAP*) gene expression [13,18-21]. Interestingly, both methods have also been reported to promote the differentiation of particular neural cell types [13,14,22-24]. To help clarify the relative influences of these different treatment conditions, direct comparisons of FBS- and CNTF-mediated differentiation are needed.

Here we employ quantitative PCR (qPCR) to examine the early sequential changes induced by these treatment conditions in the expression of a profile of genes encompassing progenitor, neuronal, and glial markers by murine RPCs. These results are in turn compared to RPC cultures treated with the same differentiating agents, but in the presence of sustained mitogenic stimulation with epidermal growth factor (EGF). This represents, to our knowledge, the first direct comparative analysis of the relative effects of these commonly employed conditions on a type of CNS progenitor cell.

## Methods

### Isolation and culture of retinal progenitor cells

RPCs were previously isolated from the neural retina of postnatal day one GFP transgenic mice [8]. Briefly, retinas of newborn GFP transgenic mice (gift from Dr. Masaru Okabe, University of Osaka, Japan) were surgically removed following decapitation and finely chopped with forceps and digested 20 min in 0.1% type I collagenase (Invitrogen, Carlsbad, CA). The supernatant with dissociated cells was then passed through a 100 μm mesh strainer and centrifuged. It was then seeded in complete culture medium, henceforth designated standard medium (SM), consisting of advanced DMEM/F12 (Invitrogen), 1% N2 neural supplement (Invitrogen), 2 mM L-glutamine (Invitrogen), 50 U/ml penicillin-streptomycin (Invitrogen), and 20 ng/ml epidermal growth factor (recombinant human EGF; Invitrogen). Cultured medium was changed every two days. GFP+ clusters (neurospheres) appeared within the first two to three days and proliferating cells were passaged at regular intervals of four to five days. These cells were immunoreactive for nestin (a marker for neural progenitor cells) and proliferation marker *K_i_-67*. All animals were handled according to ARVO animal usage standards and following approval by the animal care and use committee of the Schepens Eye Research Institute, where original derivation of the cells was performed.

### Differentiation of retinal progenitor cells in vitro

Passage 30 RPCs were trypsinized and dissociated into single cells with a fire-polished glass pipette. These cells were then plated at a density of 5×10^4^ cells/ml in T75 flasks or fibronectin-coated four-well culture slides and allowed to grow for 16–20 h at 37 °C in SM. The EGF-containing SM was then replaced with one of four alternate differentiation media still containing advanced DMEM/F12, which had 2 mM L-glutamine, 1% N2 neural supplement and 50 U/ml penicillin-streptomycin, but with different active reagents, namely, 10% FBS (Sigma-Aldrich, St. Louis, MO), 10 ng/ml CNTF (Chemicon, Temecula, CA), 10% FBS+20 ng/ml EGF, or 10 ng/ml CNTF+20 ng/ml EGF (Table 1). Controls were replated in SM. The medium was changed every two to three days. The cells were collected for RNA extraction at multiple time points, namely, day 1, day 3, day 5, and day 7. RNA was analyzed using quantitative reverse transcription (RT)–polymerase chain reaction (PCR). Additional cells were grown on four-well slides under 10% FBS conditions or in SM and fixed after seven days for immunocytochemical studies.

**Table 1:** Culture conditions used

**Treatment**	**EGF**	**CNTF**	**FBS**
Proliferation (SM)	20 ng/ml		
Differentiation			10%
		10 ng/ml	
Combination	20 ng/ml		10%
	20 ng/ml	10 ng/ml	

Media used in the experiments are illustrated here with key differences in active components shown for comparison. Proliferation conditions were based on the use of EGF-based standard medium (SM), while differentiation conditions consisted of either FBS or CNTF, at the concentrations shown, in the absence of EGF. In addition, there were 2 combination treatments used in which the mitogen EGF was combined with a differentiating agent, one example being EGF + FBS and the other EGF+CNTF, at the concentrations shown. Abbreviations: EGF represents epidermal growth factor, CNTF represents ciliary neurotrophic factor, FBS represents fetal bovine serum, SM represents standard medium. Percent is by volume.

### Cell viability assay

To assess the influence of treatment on cell viability, we tested GFP-mouse retinal progenitor cells (gmRPCs) under six different conditions consisting of baseline media and either no growth factors, 20 ng/ml EGF, 10 ng/ml CNTF, 10% FBS, 20 ng/ml EGF + 10 ng/ml CNTF or 20 ng/ml EGF +10% FBS, for three days. Cell viability was measured using the Cell Counting Kit-8 (CCK-8) assay (catalog number: CK07–11; Dojindo, Kumamoto, Japan). In brief, cells were suspended at a final concentration of 1×10^4^ cells/well and cultured in 96-well flat-bottomed microplates. After three days, 10 μl of 2-(2-methoxy-4-nitrophenyl)-3-(4-nitrophenyl)-5-(2,4-disulfophenyl)-2H-tetrazolium, monosodium salt (WST-8) was added to each well, mixed, and the plates incubated for an additional 4 h at 37 °C to convert WST-8 into formazan. The absorbance at 450 nm (OD_450_) was measured with a spectrophotometer, and the resulting OD_450_ values obtained, which were directly proportional to cell viability. All experiments were performed in triplicate.

### RNA isolation and quality controls

Total RNA was extracted from each sample using the RNeasy Mini Kit (Qiagen, Valencia, CA). DNaseI was used to digest and eliminate any contaminating genomic DNA. RNA concentration was measured for each sample at a wavelength of 260 nm (A260), and the purity of extracted total RNA was determined by the A260/A280 ratio. Quantitative RT–PCR analyses were only performed on samples that had A260/ A280 ratios between 1.9 and 2.1.

### Reverse transcription and quantitative PCR analysis

We used 2 µg of total RNA in a 20 µl reaction for reverse transcription employing an Omniscript cDNA Synthesis Kit (Qiagen). A primer set for each gene was designed using Primer3 software. The primers were synthesized commercially (Invitrogen), and qPCR was performed using an Applied Biosystems 7500 Fast Real-Time PCR Detection System (Applied Biosystems, Foster, CA) in 20 µl total volume containing 10 µl of 2× Power SYBR Green PCR Master Mix (Applied Biosystems), 10 µl of cDNA, and 300 nM of gene-specific primers (Table 2). Cycling parameters for qPCR were as follows: initial denaturation at 95 °C for 10 min, followed by 40 cycles of 15 s at 95 °C and 1 min at 60 °C. To normalize template input, we measured *β-actin* (*ACTB*) (endogenous control) transcript levels for each sample. The PCR efficiency of the reaction was measured with primers using serial dilution of cDNA (1:1, 1:5, 1:25, 1:125, 1:625, and 1:3,125). The relative expression of the gene of interest,

**Table 2:** Primers used for quantitative RT–PCR

**Genes**	**Description**	**Accession number**	**Forward (5′-3′)**	**Reverse (5′-3′)**
*Nestin*	Intermediate filament, progenitor	NM_016701	aactggcacctcaagatgt	tcaagggtattaggcaagggg
*Vimentin*	Intermediate filament, progenitor	NM_011701	tggttgacacccactcaaaa	gcttttggggtgtcagttgt
*Sox2*	Transcription factor, progenitor	NM_011443	cacaactcggagatcagcaa	ctccgggaagcgtgtactta
*Hes1*	Transcription factor, progenitor	NM_008235	cccacctctctcttctgacg	aggcgcaatccaatatgaac
*Hes5*	Transcription factor, progenitor	NM_010419	caccgggggttctatgatatt	caggctgagtgctttcctatg
*Pax6*	Transcription factor, progenitor	NM_013627	agtgaatgggcggagttatg	acttggacgggaactgacac
*Chx10*	Transcription factor, progenitor	NM_007701	caatgctgtggcttgcttta	cttgagagccactgggctac
*Mash1*	Transcription factor, progenitor	NM_008553	tctcctgggaatggactttg	ggttggctgtctggtttgtt
*Notch1*	Surface receptor, progenitor	NM_008714	acccactctgtctcccacac	gcttccttgctaccacaagc
*K_i_-67*	Cell cycle protein, proliferation	X82786	cagtactcggaatgcagcaa	cagtcttcaggggctctgtc
*β3-tubulin*	Microtubule protein, neural precursor	NM_023279	cgagacctactgcatcgaca	cattgagctgaccagggaat
*DCX*	Microtubule-associated, neuroblast	NM_010025	tgtaaactaaaacaaagacccgaag	aagtacctcacaagtcaaagaatgg
*Map2*	Microtubule-associated, neuron	NM_001039934	agaaaatggaagaaggaatgactg	acatggatcatctggtaccttttt
*Recoverin*	Phototransduction-related, retinal cells	NM_009038	atggggaatagcaagagcgg	gagtccgggaaaaacttggaata
*Rhodopsin*	Phototransduction, rod photoreceptor	NM_145383	tcaccaccaccctctacaca	tgatccaggtgaagaccaca
*PKC-α*	Bipolar neuron	NM_011101	cccattccagaaggagatga	ttcctgtcagcaagcatcac
*CRALBP*	Müller, RPE cell	NM_020599	agggtctttgttcacggagat	tgccactagagcgttcctaaa
*GFAP*	Intermediate filament, glia	NM_010277	agaaaaccgcatcaccattc	tcacatcaccacgtccttgt
*β-actin*	Housekeeping gene	NM_007393	agccatgtacgtagccatcc	ctctcagctgtggtggtgaa

Primer pairs were selected to evaluate an expression profile of 19 different murine genes. In addition to housekeeping, these included genes associated with progenitor status and proliferation, as well as neuronal, glial, and retinal lineages. This was done to enable quantitative monitoring of the relative expression levels of primitive versus mature markers, thereby allowing comparison of the relative differentiating influence of the culture conditions examined. Gene name abbreviations: Sox represents sex determining region Y-box, Hes represents hairy and enhancer of split, Pax represents paired box, Chx represents Ceh-10 homeodomain containing homolog, Mash represents mammalian achaete-scute homolog, DCX represents doublecortin, Map represents microtubule-associated protein, PKC represents protein kinase C, CRALBP represents cellular retinaldehyde binding protein.

$$\frac{{\left({\text{E}}_{\text{target}}\right)}^{\Delta \text{Ct target }(\text{control}-\text{treated})}}{{\left({\text{E}}_{\text{ref}}\right)}^{\Delta \text{Ct ref }(\text{control}-\text{treated})}}$$

was then evaluated by the Pfaffl method [25]. The value obtained from cycle threshold (C_t_) represents the number of PCR cycles at which an increase in fluorescence signal (and therefore cDNA) can be detected above background and the increase is exponential for the particular gene. Data are expressed as fold change relative to untreated controls, after normalizing to *ACTB*.

### Gene profile

The cells used in the current study have been shown to be similar in phenotypic potential to late RPCs [8,13,26]. The markers examined were therefore chosen to reflect distinguish cell types within the anticipated phenotypic spectrum (Table 2).

### Immunolabeling

RPCs were plated on four-well chamber slides coated with fibronectin. Cells were fed every two days and fixed on day 7 with freshly prepared 4% paraformaldehyde (Invitrogen) in 0.1 M phosphate-buffered saline (PBS; 2.68 mM KCI, 1.47 mM KH_2_PO_4_, 135.60 mM NaCl, 8.10 mM Na_2_HPO_4_) for 20 min at room temperature. After cells were washed in PBS, they were incubated for 1 h at room temperature in antibody blocking buffer that comprised the following: PBS containing 10% (v/v) normal goat serum (NGS; Biosource, Camarillo, CA), 0.3% Triton X-100, 0.1% NaN_3_ (Sigma-Aldrich, Saint Louis, MO). Slides were then incubated in primary antibodies (Table 3) for 24 h at 4 °C. After washing, slides were incubated for 1 h at room temperature in fluorescent-conjugated secondary antibody, either 1:500 Alexa Fluor^546^ goat anti-mouse or Alexa Fluor^546^ goat anti-rabbit (Becton Dickinson, Franklin Lakes, NJ) in PBS, followed by washings. Cell nuclei were counterstained with 1.5 µg/ml 4',6-diamidino-2-phenylindole (DAPI; Invitrogen, Molecular Probes, Eugene, OR) in Vectashield Hard Set Mounting Medium (Vector Laboratories, Burlingame, CA) for 15 min at room temperature. Negative controls for immunolabeling were performed in parallel using the same protocol but with omission of the primary antibody. Fluorescent labeling was judged as positive only with reference to the negative controls. Immunoreactive cells were visualized and images recorded using a Nikon fluorescent microscope (Eclipse E600; Nikon, Melville, NY) with excitation and emission wavelengths of fluorescence filters as follows (in nanometers): blue, excitation: 400–418, emission: 450–465; green, excitation: 465–495, emission: 515–555; red, excitation: 525–555, emission: 590–650.

**Table 3:** Primary antibodies used for immunocytochemistry

**Antibodies**	**Type**	**Specificity in retina**	**Source**	**Dilution**
Nestin	Mouse monoclonal	Progenitors, reactive glia	BD	1:200
Vimentin	Mouse monoclonal	Progenitors, reactive glia	Sigma	1:200
K_i_-67	Mouse monoclonal	Proliferating cells	BD	1:200
GFAP	Mouse monoclonal	Glia	Chemicon	1:200
β3-tubulin	Mouse monoclonal	Neurons	Chemicon	1:100
Rhodopsin	Mouse monoclonal	Photoreceptors (rods)	Chemicon	1:100
PKC-α	Rabbit polyclonal	Bipolar neurons	BD	1:200

Antibodies were selected to identify expressed protein epitopes that comprised a subset of the transcripts examined by quantitative PCR. This was done to provide confirmation at the protein level of results obtained at the level of RNA. In addition, the use of immunocytochemistry allowed examination of the relative distribution and intensity of expression levels across the cultured populations and correlation of marker expression with cellular morphology. Abbreviations: GFAP represents glial fibrillary acidic protein, PKC represents protein kinase C.

## Results

Dynamic changes in relative gene expression occurring early in the course of murine RPCs differentiation were examined. To do this, neurospheres were dissociated into single cells, plated, and grown under five culture conditions for a seven-day period. These included proliferation conditions (SM), two different differentiation conditions (FBS, CNTF), and two different combined conditions (FBS+EGF; CNTF+EGF). Morphological observations were made and gene expression data collected over the course of the experiment.

### Treatment-induced changes in RPC morphology

**Figure 1:**
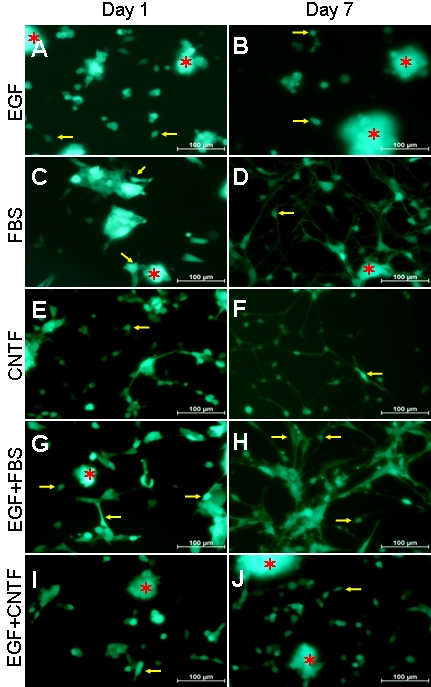
RPC treatment with different culture conditions resulted in morphological changes. RPC neurospheres were dissociated into single cells and grown under five culture conditions. In each case, some cells attached to the surface of the flask and extended processes (yellow arrows point to examples), while others formed clusters that often extended beyond the plane of focus (red asterisks mark examples). For purposes of analysis, attention was directed to the former as opposed to the latter. **A**, **B**: Under standard proliferation conditions (EGF), RPCs continued to maintain the appearance of undifferentiated neuroectodermal cells, singly or in clusters, and either adhered to the uncoated flask or floated in the culture medium. Some adherent cells extended short processes. **C**, **D**, **G**, **H**: With FBS or EGF+FBS treatment, most cells were adherent and gave out short processes by the first day, some cells becoming large with polygonal morphology **(C**, **G)**; with time, most cells exhibited two or more long processes which formed a network between cells by day 7 **(D**, **H)**, and these processes were of finer caliber with FBS treatment **(D)** than was observed with EGF+FBS **(H)**. **E**, **F:** After CNTF treatment, most cells exhibited short processes on day 1 **(E)** and formed long neurite-like processes by day 7 **(F)**. **I**, **J**: In EGF+CNTF treatment, some cells had short processes from day 1 to day 7, whereas long processes were not observed.

With time in culture, the cells exhibited increasingly divergent morphologies in response to the treatment conditions employed. Control RPCs replated in proliferation medium (SM) continued to maintain the appearance of undifferentiated progenitors, either as single cells or cellular clusters of various size, in either case adherent to the uncoated flask or floating in suspension. Characteristically, adherent cells extended only occasional short processes with few, if any, branches (Figure 1A,B). With FBS or FBS+EGF treatment, most cells extended short processes on the first day, with some cells becoming larger with polygonal morphology. Over time, more than 95% of the cells extended two or more long processes that coalesced to form an apparent network between cells by day 7. A higher density of processes was observed in FBS compared with EGF+FBS treatment (Figure 1C,D,G,H). There was a substantial attrition in cell number associated with CNTF treatment. Most of the surviving cells extended short processes at day 1 and had long, thin neurite-like processes at day 7 (Figure 1E,F). There was less cell loss associated with combined EGF+CNTF treatment, with some cells extending short processes from day 1 through day 7; however, long or thick processes were not observed (Figure 1I,J).

### Cell viability as a function of treatment condition

**Figure 2:**
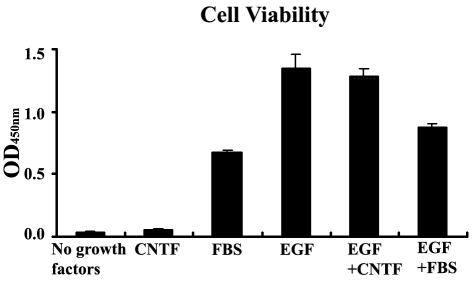
Impact of treatment conditions on RPC viability. Cells were plated at 1×10^4^ cell/well and cellular viability assessed using a formazan-based assay after three days exposure to the media compositions used in the current study. These consisted of no added growth factors, 10 ng/ml CNTF alone, 10% FBS alone, 20 ng/ml EGF alone, EGF in combination with CNT, and EGF in combination with FBS. Optical density at 450 nm is proportional to cell number. The error bars show standard deviation.

Our initial results, as described in the previous section, indicated that RPC survival was not equivalent under the different conditions and that a marked attrition in cell number was evident in medium containing CNTF (10 ng/ml) alone. To document this phenomenon and provide a direct comparison to other treatment conditions, we employed a formazan-based cell viability assay. In this assay, optical density at OD_450_ reflects the amount of reaction product in viable cells and is directly proportional to cell number (Figure 2). This assay confirmed high viability in the presence of EGF, alone (positive control) or in combination with CNTF or FBS. FBS was associated with sustained cellular viability but lower cell numbers, even in the presence of EGF. In contrast, CNTF alone was associated with markedly diminished cell numbers, approaching the low viability seen in the complete absence of added growth factors (negative control).

### Quantitative evaluation of the effect of FBS on RPC gene expression using qPCR

**Figure 3:**
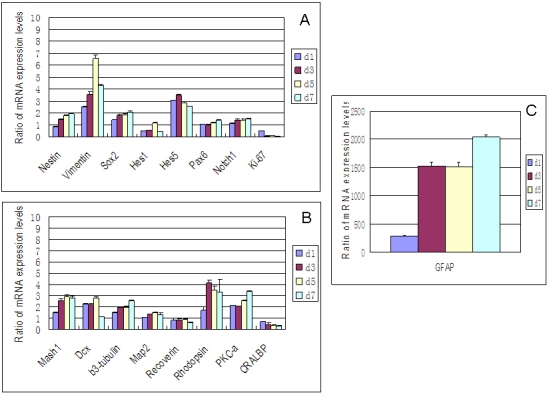
Effect of FBS on gene expression profile of RPCs by qPCR. The transcripts examined included markers associated with immaturity (**A**) *nestin, vimentin, Sox2*, *Hes1, Hes5, Pax6, Notch1,* and *K_i_-67*, as well as markers associated with lineage specification (**B**) *Mash1, DCX, β3-tubulin, Map2, recoverin, rhodopsin, PKC-α, CRALBP*, and (**C**) *GFAP*. The glial marker GFAP is presented separately because of the markedly greater expression levels. FBS treatment resulted in upregulation of most genes examined over the period of investigation, although none as substantially as *GFAP*. Abbreviations: day 1 (d1), day 3 (d3), day 5 (d5), day 7 (d7). The error bars show standard deviation.

For RPCs grown under FBS treatment conditions, most genes in the expression profile were gradually upregulated over the course of the seven-day period of observation (Figure 3A-C). Specifically, the results from qPCR (at day 7) showed a very large increase in the expression of *GFAP* (>2,000 fold), together with much smaller yet notable (>twofold) upregulation of *vimentin* (4.3 fold), *PKC-α* (3.4 fold), *rhodopsin* (*RHO*; 3.3 fold), *Mash1* (2.8 fold), *β3-tubulin* (2.6 fold), *Hes5* (2.6 fold), and *Sox2* (2.1 fold). Other genes showing marginally increased expression included *Pax6*, *Notch1*, *DCX*, and *Map2*. With FBS, expression of *K_i_-67* was markedly downregulated (28.5 fold), with smaller yet notable (>twofold) decreases in the expression of *CRALBP* (3.3 fold) and *Hes1* (2.1 fold). Recoverin (*Rcvrn*) decreased marginally.

### Quantitative evaluation of the effect of CNTF on RPC gene expression using qPCR

**Figure 4:**
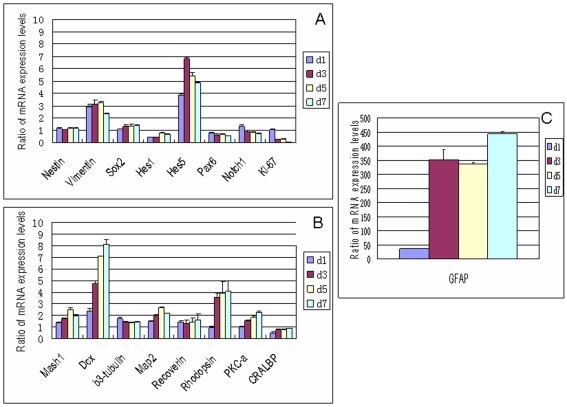
Effect of CNTF on gene expression profile of RPCs by qPCR. With CNTF treatment, expression of the proliferation marker *K_i_-67* decreased sharply while other progenitor markers were downregulated slightly or remained unchanged, with the exception of *vimentin* and *Hes5*, which increased (**A**). Most lineage and differentiation markers (**B**, **C**) were upregulated and tended to progressively increase from day 1 to day 7. Abbreviations: day 1 (d1), day 3 (d3), day 5 (d5), day 7 (d7). The error bars show standard deviation.

Following treatment with CNTF, expression of most progenitor-associated markers was downregulated slightly, or was unchanged, as compared to controls grown in SM (Figure 4A), while most precursor and differentiation markers were upregulated with relative expression levels tending to increase progressively over the seven-day course of the study (Figure 4B,C). Seven days of treatment with CNTF resulted in substantial upregulation of *GFAP* (>400 fold), although this increase was not as large as that seen with FBS treatment. There were much smaller yet notable (≥ twofold) increases in the expression of *DCX* (8.2 fold), *Hes5* (4.8 fold), *RHO* (fourfold), *vimentin* (*Vim*; 2.4 fold), *Map2* (2.2 fold), *PKC-α* (2.2 fold), and *Mash1* (twofold). Other genes displaying marginal increases in expression included *Sox2*, *β3-tubulin*, and *Rcvrn*. Expression of *K_i_-67* decreased sharply (19.6 fold). Genes with marginal decreases included *Hes1*, *Notch1*, and *CRALBP*.

### The combined effect of EGF+FBS on RPC gene expression, evaluated by qPCR

**Figure 5:**
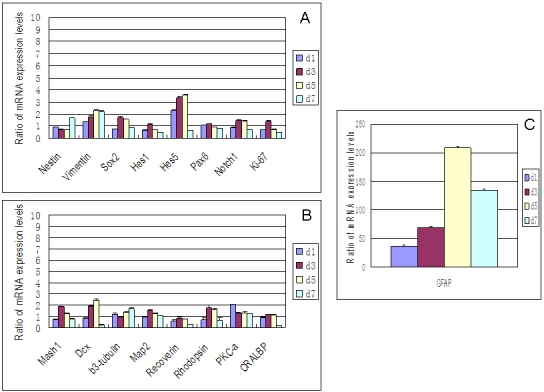
Effect of EGF+FBS on gene expression profile of RPCs by qPCR. When RPCs were treated with EGF+FBS, there was a tendency for gene expression levels to be modestly upregulated from day 1 through days 3 or 5 and then drop from peak levels by day 7 (**A**, **B**). *GFAP* was again markedly upregulated, yet also dropped from peak levels on day 7 (**C**). Abbreviations: day 1 (d1), day 3 (d3), day 5 (d5), day 7 (d7). The error bars show standard deviation.

RPCs were next treated with EGF+FBS to evaluate simultaneous exposure to both of these treatment variables. Under these conditions, 15 out of 17 genes exhibited initial increases in expression levels followed by subsequent declines from peak levels by day 7 (Figure 5). During this period, *GFAP* was strongly upregulated (>200 fold), albeit to a level approximately one order of magnitude lower than with FBS in the absence of EGF. Furthermore, *GFAP* expression peaked on day 5, and, although still quite elevated relative to controls, fell well below peak values by day 7. Other genes exhibited a similar pattern with a transient peak, albeit of much lower amplitude than that seen for *GFAP*. *Hes5* expression increased up until day 5 (3.6 fold), then dropped below control levels on day 7. *DCX* also peaked on day 5 (2.5 fold), as did *Vim* (2.3 fold). *PKC-α* peaked on day 1, then decreased. Several markers showed the same general pattern of a transient peak, although relative changes in expression were marginal as compared to controls. These included *Sox2*, *Hes1*, *Pax6*, *Notch1*, *K_i_-67*, *Mash1*, *Map2*, *Rcvrn*, and *CRALBP*. In contrast, *nestin* and *β3-tubulin* went against this trend and appeared to dip slightly on day 3 before ending higher on day 7, although the changes in expression for these markers were marginal (<2 fold). There was no notable (≥2 fold) downregulation of gene expression within the first five days of treatment compared with baseline controls. However, there were substantial drops from peak levels for many genes by day 7, as described, and a subset were notably (≥ twofold) lower than baseline expression levels at this time point, namely *CRALBP* (5.9 fold), *DCX* (3.6 fold), *Rcvrn* (3.2 fold), *Hes1* (2.1 fold), and *K_i_-67* (twofold).

### The combined effect of EGF+CNTF on RPC gene expression, evaluated by qPCR

**Figure 6:**
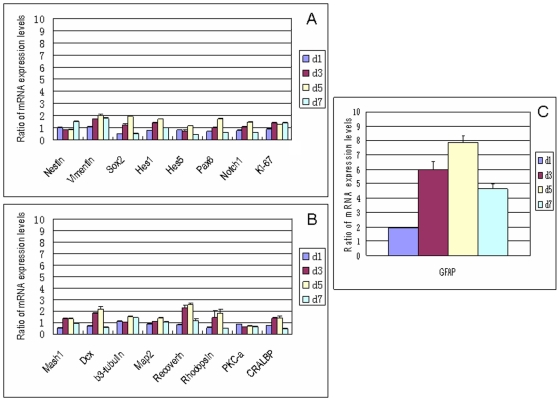
Effect of EGF+CNTF on gene expression profile of RPCs by qPCR. **A**: Expression levels of progenitor markers did not vary notably except for marginal increases in *vimentin* and *Sox2*. **B**, **C**: Except for an increase in *GFAP*, which was small compared to the data in Figure 2, Figure 3, and Figure 4, expression of lineage and differentiation markers varied only slightly, with recoverin and *DCX* showing modest increases. Abbreviations: day 1 (d1), day 3 (d3), day 5 (d5), day 7 (d7).

EGF+CNTF treatment produced results across the tested profile that tended to follow a similar temporal pattern to that seen with FBS+EGF, namely, a transient peak of increased expression, albeit in this case of lesser amplitude (Figure 6). All transcripts except *nestin* peaked before day 7, and 14 of these peaked on day 5. *GFAP* again displayed the greatest relative increase among the genes examined, however, under these conditions the peak increase was only 7.9 fold. This represents less than 2% of the maximum increase in *GFAP* expression seen with CNTF alone over the same seven-day period. None of the 16 other genes sustained a twofold or greater increase in expression, although *DCX*, *Rcvrn*, *Vim*, and *Sox2* showed transient elevations in the range of 2.0–2.5 fold. Expression of *CRALBP*, *Hes5*, and *Rho* were downregulated by 2.4, 2.1, and 2.1 fold respectively. As seen with EGF + FBS, *nestin* dipped on days 3 and 5, only to peak on day 7. Similarly *PKC-α* was again highest on day 1 and dropped from there. *β3-tubulin* again appeared to dip slightly on day 3.

### Effect of FBS on RPC marker expression, evaluated by immunocytochemistry

**Figure 7:**
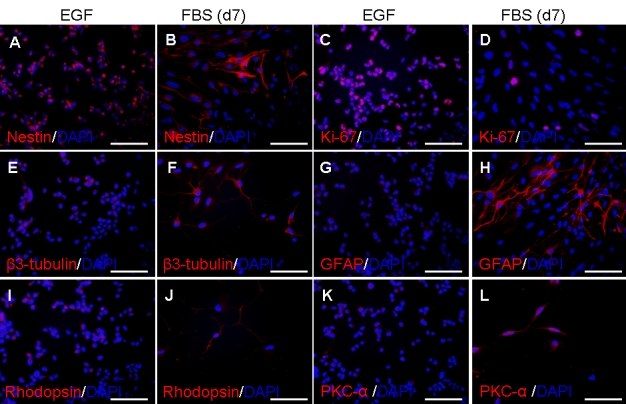
Effect of FBS on RPC expression of markers as shown by immunocytochemistry. RPCs were grown in the absence (**A**, **C**, **E**, **G**, **I**, **K**) or presence (**B**, **D**, **F**, **H**, **J**, **L**) of FBS for seven days, fixed, and immunolabeled with antibodies against *nestin* (**A**, **B**), *K_i_-67* (**C**, **D**), *β3-tubulin* (**E**, **F**), GFAP (**G**, **H**), *rhodopsin* (**I**, **J**); and *PKC-α* (**K**, **L**). Final cell densities in chamber slides were dependent upon treatment conditions. Cell nuclei were counterstained with DAPI. Scale bars represent 100 μm.

PCR detects changes in message expression, however, the extent to which these changes are reflected at the level of proteins is also of interest. Immunocytochemistry was employed for this purpose in the selected example of RPCs grown on fibronectin-coated four-well slides in the absence or presence of FBS for seven days (Figure 7). Results for protein expression were consistent with those seen at the message level under FBS treatment conditions. At baseline, over 85% of the RPCs expressed *nestin* and over 40% expressed *K_i_-67*, indicative of active proliferation. Following seven days of FBS treatment, prominent upregulation of *GFAP* was quite apparent and increased expression of other differentiation markers was evident as well, including *β3-tubulin* and *PKC-α*. A subset of treated cells exhibited rhodopsin labeling. In contrast, expression of *K_i_-67* was clearly lower with FBS. Nestin labeling was widespread in the undifferentiated cells and less so following seven days of exposure to FBS, yet was intensely expressed by a minority subpopulation (Figure 7B). This result provides a correlate for the data seen in Figure 3 in which total nestin message increased over the seven days of FBS treatment, despite the known association of nestin with undifferentiated RPCs.

Final cell densities in chamber slides were dependent upon treatment conditions (see Figure 2). Estimated percentage of cultured cells labeling for specific markers under treatment conditions are as shown (Table 4).

**Table 4:** Estimated percentage of cultured cells labeling for specific markers.

	**nestin**	**K_i_-67**	**β3-tubulin**	**GFAP**	**rhodopsin**	**PKC-α**
EGF	94	40	16	0	18 (low)	(marginal)
FBS	18	8	46	22 (high)	20 (high)	70

Immunolabeled profiles were counted under specific culture conditions and percentage of labeled cells was calculated relative to total DAPI-positive nuclei. In some instances there were striking differences in the intensity of labeling, noted here in parentheses. Abbreviations: EGF represents EGF-based proliferation conditions; FBS represents FBS-based differentiation conditions.

## Discussion

This study examines gene expression during the initial phase of RPC differentiation in vitro and reveals quantitative differences between treatment conditions, as well as dynamic changes in marker transcript levels occurring over a seven-day period. Together, these results demonstrate the utility of qPCR as a method for the investigation of dynamic changes in the gene expression of cultured RPCs under different treatment conditions. In particular, the findings of the present study call into question the notion that exposure to serum, or to defined factors such as CNTF, is entirely sufficient to reliably commit all cells of a given population to terminal differentiation. The present data indicate that continued exposure to EGF markedly diminishes the initial impact of serum and CNTF on gene expression and further undermines the influence of these reagents out to day 7, thereby implicating mitogen withdrawal as a particularly important component of differentiation protocols. This issue is not without consequence in the context of potential clinical applications, particularly in the design of protocols for the controlled differentiation of stem cells before transplantation.

It is widely appreciated that stem cells can self-renew and, potentially, be expanded in large numbers. The ultimate value of these cells, however, lies in their ability to replace mature cell types and thereby restore function to injured, diseased, or aging tissues. To accomplish this, stem cells must lose their phenotypic plasticity and undergo fate determination and differentiation. RPCs represent a subtype of the tissue-specific multipotent cells found through out the neuraxis during development [12,27-29], or even into adulthood [30], and often referred to as neural stem cells. RPCs are unique to the developing neural retina [31] and share many, but not all, of the characteristics of the equivalent stem-like cells that are found elsewhere in the central nervous system [8,32,33].

Functional differentiation following transplantation remains the essential measure of a stem cell’s therapeutic utility, as well as safety. That being the case, it is equally true that in vitro testing is considerably more efficient. Additional reasons for interest in methods of inducing differentiation in vitro include the safety advantages of differentiating stem cells in a controlled environment before transplantation, as well as pre-selection of partially differentiated cells with optimal potential for integration, as has been reported for rod photoreceptor precursor cells [9]. Therefore, the time course and relative completeness of in vitro differentiation are issues of importance, together with the phenotypic identity of the resulting cellular populations and subpopulations.

Both serum and CNTF have been used as methods of differentiating both brain- and retina-derived progenitor cells, and both of these methods are known to promote the expression of the intermediate filament *GFAP*. This marker has long been associated with glial populations, particularly mature astrocytes, but also reactive or cultured Müller cells. We have previously reported that cultured murine RPCs of the type used here express minimal, if any, *GFAP* under baseline proliferation conditions, but strongly express this marker (along with other lineage markers) after exposure to serum [8] or CNTF [13]. The present study reveals the temporal characteristics of *GFAP* induction and shows that the magnitude of *GFAP* upregulation is vastly greater than that seen for any of the neuronal lineage transcripts examined, thereby confirming the pro-glial influence of both the FBS and CNTF. That noted, immunolabeling showed that neuronal phenotypes still outnumber *GFAP*+ profiles following FBS treatment, indicating that glial specification is restricted to a subpopulation of cells under those conditions. Although a subpopulation, these nascent cells of glial phenotype are remarkable in that they were not evident in prior studies of gmRPCs transplanted to the diseased retina [8]. Furthermore, the present study was able to assess the relative efficacy of these conditions and reveals that FBS is considerable more potent in the induction of *GFAP* than is CNTF. In neither case was the mature Müller cell marker *CRALBP* upregulated over seven-day span of this study, perhaps relating to the difference in dosage when compared to other studies [23].

Beyond the powerful induction of *GFAP*, both FBS and CNTF were associated with more modest increases in transcripts for the related intermediate filament *Vim* and neuronal markers were upregulated to varying extents. The latter included the early markers *Mash1* and doublecortin (*DCX*), as well as *βIII-tubulin*, *Map2*, and the rod and bipolar markers *Rho*, *Rcvrn*, and *PKC-α*. Upregulation of *βIII-tubulin*, *Rho*, and *PKC-α*, in response to FBS was confirmed at the protein level by immunocytochemistry and, collectively, cells with these neuronal phenotypes outnumbered those expressing *GFAP*. This was particularly evident in the case of cells expressing the bipolar marker *PKC-α*. Taken together, these data are consistent with the co-induction of various neuronal subpopulations within the pool of differentiating RPCs, as previously suggested by nonquantitative methods in the setting of both FBS [8] and CNTF [13] treatment. Neither method induces a purely neuronal or glial population and attempts to source rod photoreceptor cell types at high yield will likely benefit from alternate approaches.

Concomitant to the upregulation of lineage markers, the cell cycle marker *K_i_-67* was progressively downregulated for both FBS and CNTF treatment conditions, consistent with an accumulation of post-mitotic cells during the differentiation process. This appears to relate more to the removal of EGF from the medium than to the presence of either FBS or CNTF. A more nuanced interpretation is needed for the data from markers of developmental immaturity, particularly nestin. *Nestin* is known to be highly expressed by neural stem cells, as well as the RPCs used here [8]. For that reason, *nestin* levels would be predicted to decrease under differentiation conditions, yet in the present study were modestly elevated for FBS and remained steady with CNTF. Here examination of immunocytochemical labeling (Figure 7) provides a potential explanation. As a population, FBS-treated murine RPCs lost their immature morphologies as well as widespread *nestin* immunoreactivity, however, a minority subpopulation with a more glial appearance exhibited intense *nestin* labeling and it is possible that message from these latter cells negated the overall drop in signal from the population as a whole. Of note, *nestin* is known to be upregulated in developing and reactive Müller cells [34], along with the other intermediate filament proteins *GFAP* and *vimentin*. Similarly, the *notch* signaling pathway, including the *Hes* transcription factors, are strongly expressed in neural stem cells and RPCs, but also participate in neural differentiation, specifically including gliogenesis [35].

Differentiation conditions used for neural stem/progenitor cells typically involve both the addition of an active “pro-differentiation” component (e.g., FBS or CNTF, as used here) together with discontinuation of mitogenic growth factors (e.g., EGF, as used here). Such an approach reveals little about the relative influences of these competing components of the protocol, therefore in the present study RPCs were also cultured under combined conditions in which EGF was continued, along with the addition of either FBS or CNTF. Under these conditions, the induction of *GFAP* still occurred but was muted compared to FBS or CNTF in the absence of EGF. In addition, *GFAP* message did not increase steadily over the course of the seven-day period, but peaked at day 5 for both conditions. The induction of other markers was likewise muted and frequently showed a transient peak as well. Of particular interest is the data for the proliferation marker *K_i_-67*. Whereas this marker was strongly downregulated under both differentiation conditions, it was only moderately suppressed for EGF+FBS and even somewhat elevated for EGF+CNTF.

Collectively, these data show that the overall differentiating influences of both FBS and CNTF are considerably diminished in the presence of sustained EGF stimulation. The underlying mechanism is unclear at present but might reflect a reversal of early induction or an escape of EGF-responsive subpopulations. In either case this finding does not support the notion that exposure to serum or CNTF is in itself sufficient to irreversibly commit a neural stem cell population to terminal differentiation. In contrast, cessation of stimulation with recombinant EGF is sufficient to induce changes in morphology and marker expression consistent with early differentiation. These data would serve to caution that mere exposure of cultured stem cells to “pro-differentiation” agents may be insufficient to guarantee exit from the cell cycle and that elimination of mitotic stimulation may play a more important role. Another point underscored by the current study is that cessation of mitogenic stimulation is associated with significantly decreased progenitor cell viability which can be offset by the addition of serum, but not CNTF, likely due to growth factors present in the former. Together, these findings highlight the challenges still faced when attempting to differentiate stem cells under defined, serum-free conditions. Salient to this point, a recent in vitro study has reported that embryonic stem (ES) cell-derived retinal precursors can be differentiated into photoreceptors in conjunction with sustained mitogenic FGFs, however, this process was still relatively inefficient and required lengthy time periods in culture [11]. These challenges noted, progress is being made toward the shared goal of establishing truly serum-free conditions for the differentiation of RPCs into photoreceptors and their committed precursors.
